# Promotion and economic considerations of domestic Vagus nerve stimulation within the Chinese three-tier epilepsy center system

**DOI:** 10.3389/fneur.2026.1963297

**Published:** 2026-10-21

**Authors:** Zhujing Ou, Zhijun Le, Jiayu Mi, Raowei Yan, Wenyu Liu, Enhui Zhang, Weixi Xiong, Deng Chen, Dong Zhou, Rui Xue, Xintong Wu

**Affiliations:** 1 Department of Neurology, West China Hospital of Sichuan University, Chengdu, China; 2 Financial Settlement Department, West China Hospital of Sichuan University, Chengdu, China

**Keywords:** Chinese three-tier epilepsy center system, domestic Vagus nerve stimulation, drug-resistant epilepsy, economic considerations, neuromodulation

## Abstract

**Background:**

Epilepsy affects approximately 10 million people in China, with about 30% developing drug-resistant epilepsy (DRE). Vagus Nerve Stimulation (VNS) is an established neuromodulation therapy for DRE. Domestic VNS devices completed clinical trials in 2014 and entered clinical use in 2016. This study synthesizes clinical outcomes, promotion pathways, and economic considerations of domestic VNS devices within the Chinese three-tier epilepsy center network established by the China Association Against Epilepsy (CAAE).

**Methods:**

This narrative synthesis integrates data from previously published multicenter prospective real-world studies, CAAE accreditation records, market research, and health economics literature. Clinical outcome data were derived from studies encompassing 124 adult and 87 pediatric patients with DRE across 86 epilepsy centers. A cost comparison between domestic and imported VNS devices was performed based on market data and implantation volumes from 2022–2025.

**Results:**

In adults, responder rates (≥50% seizure reduction) increased progressively from 35.4% at 1 month to 76.6% at 12 months post-implantation, with seizure-free rates reaching 10.9%. In pediatric patients, according to the available follow-up data, ≥50% responder rates (combining seizure freedom, 75% reduction, and 50% reduction) were 43.4% at visit 2, 40.6% at visit 3, 56.6% at visit 4, and 55.8% at visit 5; seizure freedom rates ranged from 1.1 to 9.3% across visits. Significant improvements were observed in sleep quality (PSQI: 5.43 → 4.44), depression (NDDI-E: 6.49 → 4.83), anxiety (GAD-7: 7.15 → 4.95), and quality of life (QOLIE-31: 54.40 → 61.33). Adverse events were mild and transient, primarily voice changes (20.3%) and cough (8.6%). The three-tier network comprises 526 centers (45 Tier-3, 259 Tier-2, 222 Tier-1) with standardized protocols and telemedicine platforms.

**Conclusion:**

Domestic VNS demonstrates encouraging efficacy, favorable safety, and improvements in comorbidities in both adult and pediatric DRE populations within the CAAE three-tier network. The network provides a structured framework for standardized application. However, the evidence is limited by the uncontrolled design of the primary studies, short follow-up, and the absence of a formal cost-effectiveness analysis. Prospective comparative clinical and economic validation is warranted.

## 1 Introduction

Epilepsy is one of the common chronic neurological disorders in China, with a prevalence of approximately 4‰–7‰, estimating around 10 million patients nationwide (1). Among these patients, about 30% develop DRE (2). These patients face not only direct health risks from seizures but also often suffer from various comorbidities such as cognitive dysfunction and mood disorders, severely impacting their quality of life (3). Traditional pharmacological treatments have limited efficacy for these patients, while surgical interventions are restricted due to factors like localization difficulties and involvement of functional areas (4–6).

VNS is indicated for patients with DRE who are not candidates for resective surgery due to multifocal or poorly localized seizure onset, involvement of eloquent cortex, or generalized epilepsy subtypes; it is also an option for patients who have failed prior epilepsy surgery or who cannot tolerate the adverse effects of antiseizure medications. Neuromodulation therefore represents an important alternative treatment option for appropriately selected patients.

VNS regulates neuronal activity in the brain by delivering electrical pulses to stimulate the cervical Vagus nerve, thereby suppressing abnormal discharges and reducing seizure frequency (7). Chinese VNS has been in clinical use since 2016. VNS offers a minimally invasive alternative to resective epilepsy surgery, requiring only a small cervical incision rather than a craniotomy (8).

Actively promoted by the CAAE, a mature Chinese three-tier epilepsy center network system has been established across the country. Through clear division of responsibilities and efficient referral mechanisms, this system optimizes the allocation of medical resources, providing institutional support and a technical foundation for the standardized application of VNS technology nationwide and for comprehensive patient management. With continuous improvement in VNS technology and the ongoing refinement of China’s epilepsy diagnosis and treatment system, by 2021, over 140 hospitals in China had implemented VNS therapy, with nearly 5,000 epilepsy patients receiving treatment (8). The proportion of domestic devices has been increasing annually. We systematically analyze the clinical application effectiveness, promotion pathways, and economic considerations of domestic VNS devices within this system, aiming to provide reference for the development of epilepsy treatment in China.

## 2 Materials and methods

### 2.1 Study design and data sources

This study is a narrative synthesis and secondary analysis that integrates data from multiple sources to evaluate the clinical outcomes, promotion pathways, and economic considerations of domestic VNS devices within the Chinese three-tier epilepsy center network. As a secondary analysis of previously published data and publicly available statistical reports, this study did not involve the collection of original patient data, human subjects, or animal experiments.

Clinical outcome data were derived from three recently published multicenter studies conducted within the Chinese three-tier epilepsy center network. The three studies are explicitly identified as: (1) Zhao et al., a prospective, multicenter, real-world study of VNS as add-on therapy in adults with epilepsy (Acta Epileptologica, 2026) (9); (2) Liu et al., a multicenter cohort study on the efficacy and safety of VNS in pediatric epilepsy in China (Acta Epileptologica, 2025) (10); and (3) Chen et al., a longitudinal study on dynamic changes in comorbid conditions following VNS for epilepsy (Acta Epileptologica, 2025) (11). We acknowledge that the adult cohort from Zhao et al. and the comorbidity cohort from Chen et al. may overlap in patients, centers, or recruitment periods. The present synthesis does not independently verify or adjust for such overlap; the findings should be interpreted with this limitation in mind.

### 2.2 Three-tier epilepsy center network data

Data on the Chinese three-tier epilepsy center network structure were obtained from CAAE accreditation records and published normative documents. The reported 526 centers (45 Tier-3, 259 Tier-2, 222 Tier-1) reflect an accreditation snapshot as of the date specified in the CAAE source document (12). Detailed source information is available in the original publication.

### 2.3 Economic data

Cost structure data comparing domestic and imported VNS devices were obtained from market research. Device implantation volume data from 2022 to 2025 were sourced from industry statistics. Long-term cost–benefit data and health economic parameters were extracted from published health economics literature on VNS therapy in Western and Chinese healthcare systems. A formal cost-effectiveness analysis (including incremental cost-effectiveness ratios, QALYs, discounting, or a decision-analytic model) was not performed; the economic evaluation is therefore a cost comparison and does not establish cost-effectiveness.

### 2.4 Device specifications

The domestic VNS system (PINS Medical, Beijing, China) received NMPA approval in 2016. Its pulse generator weighs approximately 17 g, has an expected service life of 20 years, and features a minimum programming step of 0.05 mA with rechargeable and sensing capabilities. The imported device (LivaNova, Houston, TX, United States) has an estimated battery life of 4.9–10 + years depending on stimulation parameters, with a standard programming range of 0–3.5 mA output current, 20–30 Hz frequency, and 130–1,000 μs pulse width.

## 3 Results

### 3.1 Clinical application outcomes of domestic VNS within the Chinese three-tier epilepsy center network

#### 3.1.1 Adults

A prospective real-world study covering 83 epilepsy centers nationwide enrolled 124 adult patients with DRE who received domestic VNS treatment. Over a 12-month follow-up, the responder rate (defined as ≥50% reduction in seizure frequency) was 35.4% at 1 month post-implantation, increasing to 41.6% at 3 months, 62.2% at 6 months, 64.2% at 9 months, and 76.6% at 12 months. At 1 month post-implantation, 2.4% of patients achieved seizure freedom; by 6 months, this proportion increased to 9.2%; by the end of the 12-month follow-up, the seizure-free rate reached 10.9% (9). The denominators decreased over follow-up, with 64 patients contributing at 12 months; responder rates represent available-case analyses. Loss to follow-up and reasons for attrition are reported in the original study. A repeated-measures model was not used in the present synthesis; the original study should be consulted for its analytical approach (see Table 1).

**Table 1:** Responder rate for each follow-up compared to baseline (descriptive).

Time point	Responder, *n* (%)	Non-responder, *n* (%)
Baseline	0 (0.0)	124 (100.0)
1 month	44 (35.4)	80 (64.5)
3 months	47 (41.6)	66 (58.4)
6 months	61 (62.2)	37 (37.8)
9 months	52 (64.2)	29 (35.8)
12 months	49 (76.6)	15 (23.4)

Throughout the follow-up period, the incidence of adverse events was 26.6%, primarily stimulation-related symptoms such as voice changes/hoarseness (20.3%) and cough (8.6%). Most symptoms were mild to moderate, well tolerated, and no serious adverse events requiring device removal occurred (9). Adverse events were reported over the 12-month follow-up period; the precise timing (acute post-implantation vs. persistent) is described in the original study (see Table 2).

**Table 2:** Adverse events after VNS.

Adverse events	Number (%)
No	94 (73.4%)
Yes	34 (26.6%)
Voice change or hoarseness	26 (20.3%)
Cough	11 (8.6%)
Nausea	2 (1.6%)
Headache	1 (0.8%)
Infection	2 (1.6%)
Hematoma	2 (1.6%)
Pain	1 (0.8%)

#### 3.1.2 Pediatrics

A nationwide VNS treatment study for pediatric DRE, involving 86 epilepsy centers, enrolled 87 children aged 1–16 years with refractory epilepsy and completed a 6-month follow-up assessment. The original text reported that 18.6–23.7% of children achieved a ≥ 50% reduction in seizure frequency and 11.3–17.1% achieved complete seizure freedom. However, these values are inconsistent with the visit-level data presented in Table 3. When the categories of seizure freedom, 75% reduction, and 50% reduction are combined to define ≥50% responder status, the responder rates are 18.4% at visit 1, 43.4% at visit 2, 40.6% at visit 3, 56.6% at visit 4, and 55.8% at visit 5. Seizure freedom rates are 1.1% at visit 1, 3.9% at visit 2, 4.3% at visit 3, 5.7% at visit 4, and 9.3% at visit 5. These values should be verified against the original source and corrected throughout the manuscript. Visit 1 represents the baseline assessment; visits 2–5 represent post-implantation follow-up assessments (exact time points as reported in the original study).

**Table 3:** Seizure frequency change rate for visits 2–5 compared to visit 1 (as reported in the original study).

Visit	Seizure free, *n* (%)	75% reduction, *n* (%)	50% reduction, *n* (%)	25% reduction, *n* (%)	No change, *n* (%)	Increase, *n* (%)
Visit 1	1 (1.1)	8 (9.2)	16 (18.4)	15 (17.2)	45 (51.7)	2 (2.3)
Visit 2	3 (3.9)	12 (15.8)	18 (23.7)	16 (21.1)	25 (32.9)	2 (2.6)
Visit 3	3 (4.3)	13 (18.8)	14 (20.3)	19 (27.5)	18 (26.1)	2 (2.9)
Visit 4	3 (5.7)	16 (30.2)	12 (22.6)	14 (26.4)	8 (15.1)	0 (0.0)
Visit 5	4 (9.3)	12 (27.9)	8 (18.6)	9 (20.9)	10 (23.3)	0 (0.0)

Throughout the study period, only 2 cases of mild dizziness were reported, and no serious adverse events occurred. The study also found that children who received VNS treatment earlier tended to achieve better efficacy (10). The 6-month follow-up is relatively short, and longer-term data are needed.

#### 3.1.3 Comorbidities

A longitudinal study of 113 adult epilepsy patients systematically investigated the impact of VNS on epilepsy-related comorbidities using standardized assessment tools. The results showed significant postoperative improvements in multiple areas. The Pittsburgh Sleep Quality Index (PSQI) score decreased from a baseline of 5.43 to 4.44 at the last follow-up. The Neurological Disorders Depression Inventory for Epilepsy (NDDI-E) score decreased from 6.49 to 4.83, and the Generalized Anxiety Disorder-7 (GAD-7) score decreased from 7.15 to 4.95. The Quality of Life in Epilepsy-31 (QOLIE-31) score increased from 54.40 to 61.33 (11) (see Table 4).

**Table 4:** Comparison between baseline and the last follow-up.

Result	Baseline	Last follow-up	*p*	MD
PSQI	5.43 ± 3.60	4.44 ± 3.14	<0.001	0.46–1.52
NDDI-E	6.49 ± 4.67	4.83 ± 4.37	<0.001	1.03–2.28
GAD-7	7.15 ± 5.06	4.95 ± 3.69	<0.001	1.41–3.00
QOLIE-31	54.40 ± 15.70	61.33 ± 16.19	<0.001	−9.18--4.67
No. of PWE with comorbidity	61	61		
No. of new PWE with comorbidity		8		
No. of resolve PWE with comorbidity		8	1.00	
Neurological&Phychological (N&P) comorbidity	59	60		
New onset N&P comorbidity		8		
New resolved N&P comorbidity		7	1.00	
50% respond rate for seizure		46.02%		

PSQI, Pittsburgh Sleep Quality Index; NDDI-E, Neurological Disorders Depression Inventory for Epilepsy; GAD-7, Generalized Anxiety Disorder-7; QOLIE-31, Quality of Life in Epilepsy-31; PWE, people with epilepsy; MD, mean difference.

Improvements in sleep quality, emotional well-being, and overall life satisfaction scores were already evident at the second follow-up (1 month post-implantation) when compared to baseline. Analysis using a Generalized Estimation Equation (GEE) model revealed that these improvements persisted throughout the follow-up period. Despite slight fluctuations during the last two follow-ups, there was an overall positive trend. The original study suggests that the improvement effect of VNS on comorbidities is partially independent of seizure control (11). The GEE model coefficients, covariates, effect estimates, confidence intervals, and interaction/mediation analyses are available in the source publication. The observational, single-arm design cannot establish a direct causal effect of VNS on mood, sleep, or quality of life independent of seizure improvement.

### 3.2 Development of the Chinese three-tier epilepsy center network

#### 3.2.1 Clear hierarchical collaboration and efficient resource utilization

Led by CAAE, a Chinese three-tier epilepsy center network has been established, covering 526 epilepsy centers. This system includes 45 Tier-3 epilepsy centers (comprehensive epilepsy centers), 259 Tier-2 epilepsy centers (epilepsy centers), and 222 Tier-1 epilepsy centers (epilepsy specialty clinics), providing an organizational foundation for the standardized application of VNS technology (12). This system categorizes epilepsy centers into three distinct tiers based on comprehensive standards, including infrastructure, professional teams, and diagnostic and therapeutic capabilities. Each tier undertakes different responsibilities and tasks. Regarding the nationwide standardized and homogenized promotion and application of VNS, centers at different levels assume distinct roles and responsibilities, which have been incorporated as assessment indicators into the CAAE epilepsy center accreditation documents.

Tier-3 epilepsy centers undertake tasks such as VNS surgical treatment for complex and severe cases, individualized parameter adjustment, new technology development, and professional talent training. Tier-2 epilepsy centers are responsible for the specific implementation of standardized VNS diagnosis and treatment within their regions. They conduct preoperative assessments and surgical procedures relying on multidisciplinary teams, and manage referrals to both higher and lower tiers. These centers typically also have the basic conditions to perform VNS surgery and can handle most routine VNS treatments. Tier-1 epilepsy centers undertake initial patient screening, referral coordination, as well as postoperative programming and long-term follow-up management.

To strengthen collaboration among centers at all levels, the system has established telemedicine platforms and data-sharing mechanisms (12). A VNS cloud database platform built with cloud computing and blockchain technology enables standardized management and secure sharing of electronic medical records, treatment parameters, and follow-up records. This information platform facilitates cross-institutional data access and collaborative diagnosis and treatment, and provides convenience for clinical research and quality monitoring. The system has established a regular multidisciplinary consultation mechanism, aiming to ensure that patients receive high-quality medical services regardless of their location.

#### 3.2.2 Standardized processes ensure quality

To ensure the standardization and safety of VNS treatment, CAAE organized experts to formulate a series of normative documents, including the “Chinese Expert Consensus on Vagus Nerve Stimulation for Drug-Resistant Epilepsy” (8) and the “Standard Operating Procedure (Draft) for Vagus Nerve Stimulation in Epilepsy Treatment” (13). Based on the latest domestic and international research findings and the clinical experience of multiple hospitals, these documents specify clear requirements and standards for all aspects of VNS treatment. The guidelines define the indications and contraindications for VNS therapy, specific requirements for preoperative VNS assessment, standardized steps for VNS implantation, and content for postoperative follow-up and programming.

### 3.3 Economic considerations of domestic VNS

#### 3.3.1 Cost structure comparison

There is a significant cost difference between domestic and imported VNS devices. Market research data indicates that the cost of a complete domestic VNS system is approximately 100,000 RMB, while imported devices cost around 230,000 RMB (14). The price mainly includes high-value consumables, surgery, and programming components. The data are primarily derived from high-value consumable tender data published by various provinces and municipalities, as well as approximate statistics on average surgery-related costs for patients. This price gap gives domestic devices a clear advantage in acquisition cost. The number of implantations in recent years shows that the market share of domestic devices has been steadily increasing.

Domestic device implantations increased from 900 in 2022 to 1,500 in 2024, with a projected rise to 1,800 in 2025. In contrast, imported devices remained stable (200 in 2022–2023, rising to 300 in 2024–2025). The data are sourced from market surveys and represent approximate statistics.

Beyond the inherent cost difference of the devices themselves, domestic devices may also present cost advantages in subsequent maintenance and consumable replacement. The battery life of domestic pulse generators is reported to be comparable to imported ones, yet replacement costs are lower. Domestic manufacturers usually offer faster technical support and repair services, which may reduce device downtime and indirectly lower medical costs. However, a comprehensive economic comparison should consider implantation costs, generator longevity, replacement costs, programming/follow-up costs, complication costs, technical-support expenses, hospitalization changes, and long-term healthcare utilization for both device types. These data are not fully available in the present synthesis.

## 4 Discussion

This study provides a narrative synthesis of the clinical application, promotional pathways, and economic considerations of domestic VNS devices within the established Chinese three-tier epilepsy center network. Our synthesis of data from recent multicenter studies and health economic analyses suggests that domestic VNS demonstrates sustained efficacy in reducing seizure frequency in both adult and pediatric populations with DRE, with an acceptable safety profile. It is associated with improvements in common comorbidities and quality of life. The standardized, hierarchical promotion model facilitated by the CAAE and the cost advantages of domestic devices may contribute to a scalable solution for epilepsy management in China. However, these findings should be interpreted as exploratory and require prospective comparative validation.

The clinical outcomes of domestic VNS are encouraging and broadly align with the established efficacy profile of VNS therapy reported in international literature. In the adult cohort, the responder rate progressively increased over the 12-month follow-up, reaching 76.6%. This “staircase” effect, where efficacy improves over time, is a well-documented phenomenon in VNS therapy, attributed to gradual neuromodulatory changes in brain networks (15). The adult responder rate in our synthesis is numerically higher than that reported in some international multicenter studies. For example, the CORE-VNS international registry reported a responder rate of approximately 50.5% at ≥5 years with 13.1% seizure-free, and a recent multicenter cohort study from China reported a responder rate of 60.0% at 2 years with 15.0% seizure-free. A systematic review and meta-analysis reported an overall responder rate stabilizing at 61.8% at ≥21 months. The higher responder rate in our cohort may reflect differences in patient selection, follow-up definitions, or the “staircase” effect being captured at a relatively early follow-up point. A comparison of key studies is provided in Table 5.

**Table 5:** Comparison of VNS outcomes across selected studies.

Study	Design	*N*	Follow-up	Responder rate (≥50% reduction)	Seizure-free rate
Present synthesis (adults)	Secondary analysis	124	12 months	76.60%	10.90%
Zhao et al. (9) (China)	Prospective, multicenter, real-world	124	12 months	76.60%	10.90%
CORE-VNS international registry	Prospective observational	>500	≥5 years	~50.5%	13.10%
Multicenter cohort study (China)	Retrospective	~95	≥2 years	60.00%	15.00%
Systematic review/meta-analysis	Meta-analysis	—	≥21 months	61.80%	—

The pediatric data, while demonstrating a more modest overall responder rate, are clinically relevant. The finding that earlier VNS intervention in children tended to yield better efficacy is a potentially important clinical insight, supporting consideration of earlier VNS in the treatment pathway for pediatric DRE (16). However, the 6-month follow-up is substantially shorter than the adult follow-up, and given the known slower neuromodulatory response in children with developmental and epileptic encephalopathies, the pediatric responder rates reported here should be interpreted as preliminary. Direct comparisons between adult and pediatric efficacy endpoints should be made with caution. The lower responder rate compared to adults may be attributed to differences in the underlying etiology of epilepsy in children, which often includes more severe and diffuse developmental and epileptic encephalopathies (17). The safety profile was consistent across both adult and pediatric studies, with the most common adverse events being stimulation-related and transient, such as voice changes and cough. The absence of serious adverse events requiring device removal suggests favorable tolerability of domestic VNS. These findings are consistent with the well-established safety profile of VNS and support the manufacturing quality and clinical application protocols for the domestic device (26).

Beyond seizure control, the significant improvements in sleep quality, depressive symptoms, anxiety, and overall quality of life represent an important finding. The original study suggests that these improvements are partially independent of seizure control. It supports the hypothesis that VNS may exert a direct and positive effect on the neurobiological substrates of mood, sleep, and cognition, possibly through its influence on the locus coeruleus and other brainstem nuclei involved in the noradrenergic and serotonergic systems (18). However, the observational, single-arm data demonstrate associations over time and cannot by themselves establish a direct causal effect independent of seizure improvement. The dual benefit, controlling seizures and improving comorbidities, positions VNS as a potentially valuable therapy in the comprehensive management of DRE, but causal mechanisms require further investigation.

The success of domestic VNS cannot be understood in isolation from the healthcare infrastructure that supports it. The Chinese three-tier epilepsy center network is an important structural element. It provides a structured, efficient, and standardized framework that addresses a critical bottleneck in epilepsy care in China, the uneven distribution of specialist expertise. This model aims to ensure that complex and expensive treatments like VNS are not concentrated only in major metropolitan centers. The clear division of labor within the network is particularly notable. Tier-3 centers focus on complex cases, training, and innovation, preserving their capacity for high-level care. Tier-2 centers act as regional hubs, performing most routine VNS implantations and management, thereby increasing accessibility for a broader patient population. Tier-1 centers are crucial for patient triage, referral, and long-term follow-up, which is essential for the chronic management of VNS therapy. This hierarchical model, combined with telemedicine platforms and data-sharing mechanisms, may help mitigate the challenges of a vast and populous country. It aims to ensure that patients can be initially evaluated and followed up at local centers (Tier-1) while being referred to higher-tier centers for surgery or complex programming. The VNS cloud database, leveraging modern technology, is intended to facilitate data-driven quality monitoring and clinical research, creating a learning healthcare system. This coordinated approach stands in contrast to uncoordinated healthcare systems, where patients with complex conditions often navigate a fragmented landscape, leading to delays in treatment and inefficient resource use (19, 20). However, no comparative or longitudinal analysis demonstrates that the network itself caused increased access or improved outcomes; these remain descriptive health-system observations.

The substantial cost difference between domestic and imported VNS devices provides an economic rationale for considering domestic technology. In a country with a large population and an annual incidence of 400,000 new epilepsy cases, even a modest increase in the uptake of VNS among eligible DRE patients could translate into significant national healthcare cost savings. The economic burden of uncontrolled epilepsy is immense, encompassing direct medical costs (frequent emergency visits, hospitalizations), indirect costs (lost productivity, caregiver burden), and intangible costs (reduced quality of life) (21–23). The growing market share of domestic devices may reflect increasing acceptance within the medical community. This trend is likely driven by the convergence of evidence regarding clinical efficacy and cost advantages. The lifecycle cost advantage, including lower replacement costs and faster technical support, further strengthens the economic case for domestic devices. By making VNS therapy more affordable, China may be taking a step toward improving access to advanced neuromodulation. However, the price differential is also influenced by country-specific policies and taxes. Import tariffs (approximately 3%) and value-added tax (13%) are levied on imported medical devices in China. Under the Hainan Free Trade Port zero-tariff policy, eligible medical institutions may import approved medical devices exempt from import duties and VAT. Both domestic and imported VNS devices have obtained regulatory approval from their respective authorities (NMPA for domestic devices; FDA and CE for imported devices). The domestic VNS was approved by the NMPA for clinical use in 2016, while imported devices have been available internationally for over two decades. These regulatory and policy factors should be considered when interpreting the economic comparison.

The Chinese experience, as detailed in our study, may offer a potentially replicable model for other low- and middle-income countries, which bear a disproportionately high burden of epilepsy (24). This approach is built on three interconnected strategies: the development and clinical validation of a cost-effective domestic VNS device, the establishment of a nationwide, tiered network that standardizes care, leverages economies of scale, and optimizes resource allocation, and the proactive role of a national professional association in creating evidence-based guidelines, training the workforce, and certifying centers of excellence. This integrated approach could be adapted by other nations seeking to integrate neuromodulation into their epilepsy care pathways. With strong leadership, strategic planning, and a commitment to domestic innovation, it may be possible to build a sustainable and scalable healthcare solution for complex chronic diseases. However, the evidence base is limited, and prospective comparative clinical and economic validation is required before broad claims of replicability can be made.

Despite the potential achievements of domestic VNS within the Chinese three-tier epilepsy center network, several challenges remain. Firstly, the issue of uneven technological adoption across regions persists. Data shows that the number of epilepsy centers in eastern China accounts for 43.91% of the national total, while the northeastern region accounts for only 4.83%. The volume of VNS treatments is also highly concentrated in eastern regions. In the cumulative VNS procedures performed from 2022–2023, eastern China accounted for 66.47%, while western and northeastern regions accounted for less than 15%, with treatment gaps still existing in areas like Tibet and Inner Mongolia (25). This geographical imbalance reflects, to some extent, the regional concentration of medical resources in China, where economically developed and densely populated areas possess more medical resources and service capabilities.

The VNS operational capacity of primary healthcare institutions needs to be enhanced. Survey data in 2023 indicates that among 435 epilepsy centers nationwide, 45 Tier-3 centers (10.34%) accounted for 66.37% of the VNS treatment volume, while the more numerous Tier-2 centers had relatively limited treatment volumes (25). This suggests that the potential of Tier-2 centers for implementing VNS has not been fully realized. Future efforts should strengthen the construction of Tier-2 epilepsy centers. Through systematic training and academic exchanges, their VNS diagnosis and treatment capabilities should be improved, enabling them to undertake more VNS treatment tasks.

The present study has the following limitations. As a narrative synthesis and secondary analysis, it is reliant on the quality and design of the primary studies. The primary studies included were either prospective real-world cohorts or single-arm studies without a control group. The lack of a sham-control or a direct comparator group limits the ability to definitively attribute all observed effects solely to VNS, as some improvements may be attributable to the placebo effect or regression to the mean. This limitation is a common characteristic of most VNS clinical studies. The follow-up periods for the primary studies were relatively short, with the adult cohort having a maximum of 12 months and the pediatric cohort only 6 months. While the progressive improvement in outcomes is encouraging, longer-term data (e.g., 2–5 years) are needed to confirm the durability of the clinical response and to better understand long-term safety and battery longevity issues. The economic analysis is based on market research and health economic parameters from the literature; a more robust, prospectively collected cost-effectiveness analysis would be beneficial to precisely quantify the net cost savings and quality-adjusted life years (QALYs) gained with domestic VNS compared to imported devices or other treatments. The present economic evaluation does not report incremental cost-effectiveness ratios, QALYs, health-state utilities, discounting, or a formal economic model, and therefore should be considered a cost comparison rather than a cost-effectiveness analysis. Finally, because the study is based on data from centers within the CAAE network, it may not fully represent the outcomes of VNS therapy in smaller, non-accredited centers, potentially creating a selection bias. There is also a lack of direct clinical comparison between domestic and imported VNS devices; the domestic device cannot be assumed to have equivalent clinical effectiveness to imported devices unless comparative evidence supports that conclusion.

## 5 Conclusion

Within the Chinese three-tier epilepsy center network, domestic VNS has achieved standardization in technology application, informatization in management, and optimization in resource allocation. Clinical studies suggest that domestic VNS demonstrates encouraging efficacy and safety in both adult and pediatric patients with refractory epilepsy, and its effectiveness may show cumulative enhancement over time. It also appears to improve patients’ comorbid conditions and quality of life. However, these conclusions are based on uncontrolled, short-term studies and should be considered preliminary.

Through the optimized resource allocation and efficiency gains of the Chinese three-tier epilepsy center network, domestic devices show potential advantages in multiple dimensions, including initial device investment, operational efficiency, and long-term benefits, highlighting their clinical application and economic value. However, formal cost-effectiveness analysis and prospective comparative clinical validation are needed before these advantages can be confirmed.

The core value of this model lies in the integration of technological innovation and system construction. Through standardized diagnostic and therapeutic processes, informatized management platforms, and systematic quality control systems, it aims to ensure that new technologies can be applied normatively and effectively across medical institutions at different levels. This model may provide a reference for the long-term diagnosis and treatment management of other chronic diseases. Nonetheless, the present evidence is exploratory and requires prospective comparative clinical and economic validation.

## Data Availability

The raw data supporting the conclusions of this article will be made available by the authors, without undue reservation.

## References

[1] Ding D Zhou D Sander JW Wang W Li S Hong Z. Epilepsy in China: Major Progress in the past two decades. Lancet Neurol. (2021) 20:316–26. doi: 10.1016/s1474-4422(21)00023-5, 33743240

[2] Kwan P Arzimanoglou A Berg AT Brodie MJ Allen Hauser W Mathern G . Definition of drug resistant epilepsy: consensus proposal by the ad hoc task force of the Ilae commission on therapeutic strategies. Epilepsia. (2010) 51:1069–77. doi: 10.1111/j.1528-1167.2009.02397.x, 19889013

[3] Monteagudo-Gimeno E Sánchez-González R Raduà-Castaño J Fortea-González L Boget-Llucià T Carreño-Martínez M . Association between depressive and anxious symptoms with cognitive function and quality of life in drug-resistant epilepsy. Heliyon. (2023) 9:e20903. doi: 10.1016/j.heliyon.2023.e20903, 37886767 PMC10597766

[4] Jehi L. Advances in therapy for refractory epilepsy. Annu Rev Med. (2025) 76:389–402. doi: 10.1146/annurev-med-050522-034458, 39532109

[5] Merenzon MA Sivaraju A Herlopian A Gerrard JL Quraishi IH Hirsch LJ . Combined neuromodulation and resection for functional cortex epilepsy: a case series. J Neurosurg. (2024) 141:1527–35. doi: 10.3171/2024.3.Jns24122, 38848588

[6] Khoo A de Tisi J Mannan S O'Keeffe AG Sander JW Duncan JS. Reasons for not having epilepsy surgery. Epilepsia. (2021) 62:2909–19. doi: 10.1111/epi.17083, 34558079

[7] Smalley E. The business of brain-computer interfaces. Nat Biotechnol. (2019) 37:978–82. doi: 10.1038/s41587-019-0231-y, 31406328

[8] Wu Y Zhou J Guan Y Meng F Ji T. Chinese expert consensus on Vagus nerve stimulation for drug-resistant epilepsy. J Epilepsy. (2021) 7:191–6.

[9] Zhao C Zhang E Zhang H Yan R Le Z Chen Y . Vagus nerve stimulation as an add-on therapy in patients with epilepsy: a prospective, multicenter, real-world study in China. Acta Epileptol. (2026) 8:30. doi: 10.1186/s42494-026-00272-4, 42581390 PMC13459777

[10] Liu W Li W Zhao C Wu X Zhou D. The efficacy and safety of Vagus nerve stimulation in Pediatric epilepsy in China: a Multicenter cohort study. Acta Epileptol. (2025) 7:49. doi: 10.1186/s42494-025-00221-7, 41327507 PMC12670821

[11] Chen D Zhu L Liu L Zhou D Wu X. Dynamic changes in comorbid conditions following Vagus nerve stimulation for epilepsy. Acta Epileptol. (2025) 7:33. doi: 10.1186/s42494-025-00222-6, 40448234 PMC12123979

[12] Chinese Anti-Epilepsy Association. Standardized procedure promotion and application of Vagus nerve stimulation for epilepsy under Chinese three-tier epilepsy Center system. J Epilepsy. (2025) 11:93–5.

[13] Wu X Zhao C Zhou D. Standard operating procedure for Vagus nerve stimulation in epilepsy (draft). J Epilepsy. (2025):1–5.

[14] Helmers SL Duh MS Guérin A Sarda SP Samuelson TM Bunker MT . Clinical and economic impact of Vagus nerve stimulation therapy in patients with drug-resistant epilepsy. Epilepsy Behav. (2011) 22:370–5. doi: 10.1016/j.yebeh.2011.07.020, 21872534

[15] Scarabello A Zanuttini L Schettino M Muccioli L Ferri L Bisulli F . The impact of Vagus nerve stimulation on the Most disabling seizures: a retrospective study in adults with drug-resistant epilepsy. Neurol Sci. (2026) 47:247. doi: 10.1007/s10072-026-08868-x, 41663857 PMC12886259

[16] Kannan S Somanathan S Suvarna R Weerasooriya MM Vakharia VN. Factors predicting the response to Vagus nerve stimulation (Vns) in Pediatric patients with drug- resistant epilepsy: a systematic review and Meta-analysis. Childs Nerv Syst. (2025) 41:423. doi: 10.1007/s00381-025-07080-0, 41400737

[17] Holmes GL. Epilepsy in the developing brain: lessons from the laboratory and clinic. Epilepsia. (1997) 38:12–30. doi: 10.1111/j.1528-1157.1997.tb01074.x, 9024181

[18] Conway CR Xiong W. The mechanism of action of Vagus nerve stimulation in treatment-resistant depression: current conceptualizations. Psychiatr Clin North Am. (2018) 41:395–407. doi: 10.1016/j.psc.2018.04.005, 30098653

[19] Carpio A Hauser WA. Epilepsy in the developing world. Curr Neurol Neurosci Rep. (2009) 9:319–26. doi: 10.1007/s11910-009-0048-z, 19515285

[20] Singh G Sharma M Krishnan A Dua T d'Aniello F Manzoni S . Models of community-based primary Care for Epilepsy in low- and middle-income countries. Neurology. (2020) 94:165–75. doi: 10.1212/wnl.0000000000008839, 31919114

[21] Begley CE Durgin TL. The direct cost of epilepsy in the United States: a systematic review of estimates. Epilepsia. (2015) 56:1376–87. doi: 10.1111/epi.13084, 26216617

[22] Strzelczyk A Reese JP Dodel R Hamer HM. Cost of epilepsy: a systematic review. PharmacoEconomics. (2008) 26:463–76. doi: 10.2165/00019053-200826060-00002, 18489198

[23] Toledo M Smeyers Durá P González Martínez F Behzadi Koochani N Ciordia Dominguez R Saborido CM . The burden of seizure emergencies in Spain: a systematic literature review. Epilepsy Behav. (2026) 181:111056. doi: 10.1016/j.yebeh.2026.111056, 42068609

[24] Collaborators GE. Global, regional, and National Burden of epilepsy, 1990-2016: a systematic analysis for the global burden of disease study 2016. Lancet Neurol. (2019) 18:357–75. doi: 10.1016/s1474-4422(18)30454-x, 30773428 PMC6416168

[25] Yan R Zhao C Zhou D Zhang J Zhang K Wu Y . Survey on the current status of Vagus nerve stimulation for epilepsy under Chinese three-tier epilepsy Center system. J Epilepsy. (2025) 11:226–30.

[26] Geng G Hu W Meng Y Zhang H Zhang H Chen C . Vagus nerve stimulation for treating developmental and epileptic encephalopathy in young children. Front Neurol. (2023) 14:1191831. doi: 10.3389/fneur.2023.1191831, 37928141 PMC10624125

